# Intelligence

**Published:** 1831-09

**Authors:** 


### INTELLIGENCE.

---

### ROYAL COLLEGE OF SURGEONS, LONDON.

*Collegial Triennial Prize.* The Board of Curators announce that the subject for this prize is "on the Effects produced by complete or partial Division of the various Nerves, or by other Injuries to those Structures." Candidates to be members of the College, not of the council. Dissertations to be in English, and to be distinguished by a motto or device, accompanied by a sealed paper, containing the name and residence of the author, and having on the outside a motto or device corresponding with that on the dissertation. Dissertations, addressed to the secretary, to be delivered to the College before Christmas-day 1833.

*Jacksonian Prize.* Two prize subjects are proposed for the year 1832, viz. "The Symptoms occasioned by the different Mineral Poisons when taken into the Stomach; the proper Antidotes and Treatment which Experience or Science affords for each; and when the event is fatal, the Appearances or Effects which are generally found in the Dead Body;" and "The Mode of Union of Simple and Compound Fractures." Dissertations to be addressed to the secretary, and delivered at the College before Christmas-day 1832.

The prize subjects for the present year, 1831, are "The History and Treatment of Fractures of the Ribs, of the Sternum, and of the Pelvis, together with their Influence on the adjacent Viscera;" and "Diseases of the Eye and its Appendages, and the Treatment of them." Dissertations to be delivered at the College before Christmas-day next.

---

### APOTHECARIES' HALL.

*To the Editor of the London Medical and Physical Journal.*

Sir: I am instructed by the Court of Examiners to inform the Editors of the Medical Journals, that the names of gentlemen to whom the Court grant certificates of qualification, on each day of examination, will in future be placed in the beadle's office, on the following morning, and that each list of names will remain for public inspection (during office hours) for the space of one week.

I have the honour to be, sir,
Your obedient servant,

*Apothecaries' Hall; August* 4, 1831. JOHN WATSON.

---

### KING'S COLLEGE, LONDON.

Octo The ceremony of opening King's College will take place on Saturday, the 8th of [redacted]ber, when the Bishop of London will deliver the inaugural discourse. On the following Monday the medical school will open.

Medical students will have the option of attending the Lectures at King's College in one of two ways; either as *Occasional Pupils*, or as *King's College Medical Students.* As occasional pupils, they will be at liberty to enter to what courses of lectures they please, pledged to no special conditions, and enjoying therefore no privileges. The terms of attendance are much the same as have hitherto been adopted by the leading schools in the metropolis. King's College students, on the other hand, have the privilege of contending for prizes, which will be annually offered for superior proficiency in medical studies. Some difference is likewise made in their favor in the terms of attendance. They are expected to enter, at King's College, to all the courses of lectures upon which the College of Surgeons and Society of Apothecaries require certificates of attendance, before granting their respective diplomas: they are expected further to attend divine service every Sunday in the forenoon, in the College chapel.

We understand that no doubt is entertained that King's College, having a royal charter, will confer medical diplomas. We hail with great pleasure the establishment in the metropolis of a chartered college: it has long been a reproach to London, that alone, among the capitals of Europe, she has neglected to foster and promote, by means of collegiate institutions, the education of her sons.

### Dr. Barry's *Account of Cholera.*

We subjoin an extract of a letter from Dr. Barry, which contains the most striking and graphic description of cholera which has yet fallen under our notice:

*St. Petersburgh; July* 20, 1831.

The disease is certainly somewhat mitigated, both as to the number and the fatality of attacks, though the weather has continued unchanged. Thermometer in our apartment steadily above 70° of Fahrenheit; very little wind, and what there is constantly from the east, with the exception of about twenty-four hours last week.

Names for diseases or medicines, so contrived as to constitute little definitions, are bad things. I came here with an impression strongly fixed upon my mind, that the essential and dangerous features of cholera morbus were immoderate and ungovernable vomiting and purging of a serous fluid, violent spasms, and the exhaustion and collapse necessarily attendant on such a state; consequently, that the first indication would be to restrain these depressing evacuations. The fact is, however, that vomiting and purging are amongst the least important symptoms of the present epidemic, though the appearance of the fluid evacuated is highly characteristic. Rice water strained and allowed to settle down, is, when shaken up, the best type. The evacuations, both upwards and downwards, either soon cease or are easily repressed; while in many cases, and these the very worst, there are either none, or they are very trifling. It is the sudden paralysis and rapidly diminishing action of the heart, of the arteries, and of the organs of respiration, with the stasis and thickening of the blood, the loss of the power to generate heat, that constitute the real danger of the first, the most fatal stage of this disease. Blue, black, flat lines, mark the course of the larger veins; a deadly livor colours the skin; even the tongue is icy cold; the respiration is short, quick, and imperfect; the scrobiculus cordis and diaphragm drawn violently upwards and inwards; the pulse and voice extinct; the limbs and belly torn with spasms; the hands and feet shrivelled, corrugated, and much diminished in volume; the reason unimpaired. It would seem as if all the colourless cells and vessels, upon which the turgor or plumpness of the integuments so much depends, were squeezed to emptiness, and nothing left but the thickened colouring matter of the blood. If this state cannot be overcome in a very few hours, the sufferer must die. *Mordechi*, or *mort de chien*, or *mort noir*, would, either of them, be a much more appropriate name for this inexplicable malady, than that by which it is at present designated. I am now quite convinced that neither Celsus nor Sydenham ever saw this disease, else they surely would not have omitted *all* the symptoms that I have just enumerated.

It is in the above state, particularly if there be violent spasm, that the Magisterium bismuthi has been found so serviceable, assisted by cordials, sinapisms covering the whole belly, and frictions. Neither warm baths nor vapour baths will do: the body is warmed by them, as a dead animal would be, but the faculty of generating heat not being restored, the patient cools down rapidly again, and with increased debility. Opium appears really to be contra-indicated, unless to allay vomiting and spasm, which the bismuth effects much better; and calomel they have not ventured to give in large doses.

Two physicians, (Germans,) Ysenbeck and Brailow, stated publicly and firmly yesterday, in my presence, at the Medical Council, that during the preceding eleven days they had treated, at the Customhouse Hospital, thirty cholera patients, of whom they *had not lost one*. They give two tablespoonfuls of common table salt in six ounces of hot water, at once; and one tablespoonful of a similar mixture, cold, every hour afterwards. They always begin by bleeding.

But in the ordinary way of treating the disease: suppose the first stage safely past; very rarely indeed (not five times in the hundred) does the patient return to health without passing through a dangerous fever, which not unfrequently assumes a typhoid character, with reddish-brown dry tongue; stupor; suffused eye; constipated and tender belly; dark sordes about the lips and teeth. The pulse, however, is generally quicker, and the skin hotter, than in primitive typhus. In this state many die from the fourth to the seventh day, and even later. In other cases the fever is benignant, and goes off within the fourth day by copious perspiration.

My object in entering into this detail is to warn you that many, and fatal, cases of the present epidemic, may occur with little or no vomiting or purging. The shrivelling of the fingers and toes, the colour of the skin, the shrinking of the features, the coldness of the tongue, the feebleness or extinction of the pulse and voice, the rice-water evacuations, when there are any, are the true marks of the disease, not to be mistaken.—*Med. Gazette.*

---

### BIBLIOGRAPHICAL NOTICES.

### LITERARY INTELLIGENCE.

Dr. Morton will very shortly publish a small octavo volume, to be entitled "Remarks on the Subject of Lactation; containing Observations on the healthy and diseased Conditions of the Breast-milk; the Disorders frequently produced in Mothers by Suckling; and numerous illustrative Cases, proving that, when protracted, it is a common Cause in Children of Hydrencephalus, or Water in the Brain, and other serious Diseases."

Preparing for publication, early in September, a third edition of Dr. Ryan's "Manual of Midwifery," considerably improved and enlarged; with numerous plates.

In the press, and nearly ready for publication, in one volume octavo, the Second Edition, with a copious Supplement, of "An Essay on the Epidemic Cholera of India," by Reginald Orton, Surgeon, H.P., late of his Majesty's thirty-fourth Regiment of Foot."

### METEOROLOGICAL JOURNAL,

By Messrs. Harris and Co. Mathematical Instrument Makers, 50, High Holborn.

| | Moon. | Rain gauge. | Thermom. | | | Barometer. | | De Luc's Hygrometer. | | Winds. | | Atmospheric Variation. | | |
|---|---|---|---|---|---|---|---|---|---|---|---|---|---|---|
| *July* | | | *9 a.m.* | *max.* | *min.* | 9 *a.m.* | 10 *p.m* | 9 *a.m.* | 10*p.m.* | 9 *a.m.* | 10*p.m.* | 9 *a. m.* | 2 *p.m.* | 10 *p.m.* |
| 20 | | | 65 | 66 | 55 | 29.43 | 29.54 | 55 | 51 | SW | SW | Cloudy | Fine | Cloudy |
| 21 | | | 63 | 67 | 57 | .62 | .66 | 48 | 48 | WNW | W | Fine | — | — |
| 22 | | .24 | 61 | 64 | 55 | .63 | .63 | 50 | 50 | WNW | WSW | — | Rain | Fine |
| 23 | | | 61 | 65 | 57 | .60 | .54 | 51 | 52 | SW | ESE | — | Cloudy | Cloudy |
| 24 | ○ | .28 | 62 | 66 | 62 | .63 | .79 | 52 | 51 | ENE | ENE | — | Fine | Fine |
| 25 | | | 67 | 71 | 59 | .85 | .91 | 50 | 50 | NE *v.* | SE | — | — | — |
| 26 | | | 68 | 75 | 62 | .95 | .99 | 50 | 50 | ENE | SSE | — | — | — |
| 27 | | | 68 | 75 | 66 | 30.02 | 30.00 | 50 | 50 | WSW | NNW | — | — | — |
| 28 | | | 71 | 72 | 65 | 29.96 | 29.89 | 50 | 52 | N | ESE | — | — | — |
| 29 | | | 72 | 74 | 61 | .91 | .95 | 52 | 60 | ESE | SSE | — | Cloudy | Cloudy |
| 30 | | | 69 | 71 | 62 | .97 | .92 | 58 | 57 | NE | ESE | Cloudy | Fine | Fine |
| 31 | | | 70 | 73 | 60 | .90 | .89 | 54 | 48 | ENE | E | Fine | — | — |
| *August* | | | | | | | | | | | | | | |
| 1 | ☾ | .31 | 70 | 70 | 64 | .84 | .74 | 50 | 52 | NE | N | — | — | Rain |
| 2 | | | 69 | 64 | 62 | .64 | .68 | 55 | 58 | N | NNE | Cloudy | Cloudy | Fine |
| 3 | | .22 | 64 | 68 | 62 | .68 | .66 | 65 | 67 | E | ESE | Rain | Fine | — |
| 4 | | .17 | 65 | 72 | 66 | .58 | .51 | 67 | 65 | ESE | ESE | Cloudy | — | — |
| 5 | | | 70 | 71 | 61 | .46 | .47 | 62 | 61 | W | WSW | Cloudy | — | Cloudy |
| 6 | | .40 | 67 | 69 | 58 | .53 | .51 | 60 | 54 | WSW | SSW | Rain | — | Fine |
| 7 | ● | | 64 | 67 | 61 | .55 | .57 | 56 | 56 | | NNE | — | Cloudy | — |
| 8 | | | 65 | 71 | 65 | .62 | .68 | 59 | 57 | NE | NNW | — | Cloudy | — |
| 9 | | | 70 | 75 | 64 | .72 | .74 | 53 | 51 | W | SW | — | — | Rain |
| 10 | | | 68 | 71 | 62 | .83 | .92 | 50 | 50 | NNW | NNE | — | Fine | Fine |
| 11 | | .20 | 66 | 69 | 63 | .97 | .94 | 50 | 49 | NE | SE | — | — | — |
| 12 | | | 68 | 78 | 66 | .94 | .89 | 49 | 50 | SE | NE | — | — | — |
| 13 | | | 69 | 74 | 59 | .85 | .81 | 51 | 50 | NE | NE | — | Rain | — |
| 14 | | | 67 | 71 | 58 | .81 | .85 | 69 | 50 | NNE | NE | — | Fine | — |
| 15 | ☽ | .16 | 64 | 71 | 60 | .88 | .91 | 52 | 53 | ENE | ESE | — | — | — |
| 16 | | | 63 | 68 | 62 | .93 | .90 | 53 | 53 | ESE | NW | Rain | — | Cloudy |
| 17 | | | 66 | 72 | 60 | .85 | .78 | 53 | 54 | NNW | NW | Cloudy | — | Fine |
| 18 | | | 65 | 68 | 57 | .73 | .72 | 54 | 55 | N | NW | Fine | — | Cloudy |
| 19 | | .26 | 63 | 69 | 59 | .47 | .52 | 55 | 53 | WSW | NW | Cloudy | Cloudy | Fine |

The quantity of Rain fallen, in the month of July, was 2 inches and 56.100ths.

